# Identification of Free-Living and Particle-Associated Microbial Communities Present in Hadal Regions of the Mariana Trench

**DOI:** 10.3389/fmicb.2016.00665

**Published:** 2016-05-09

**Authors:** Jonathan Tarn, Logan M. Peoples, Kevin Hardy, James Cameron, Douglas H. Bartlett

**Affiliations:** ^1^Marine Biology Research Division, Scripps Institution of Oceanography, University of CaliforniaSan Diego, La Jolla, CA, USA; ^2^Global Ocean Dynamics, Global Ocean DesignSan Diego, CA, USA; ^3^Avatar Alliance FoundationEncino, CA, USA

**Keywords:** dark biosphere, deep-sea microbiology, hadal, marine microbial diversity, piezophile

## Abstract

Relatively few studies have described the microbial populations present in ultra-deep hadal environments, largely as a result of difficulties associated with sampling. Here we report Illumina-tag V6 16S rRNA sequence-based analyses of the free-living and particle-associated microbial communities recovered from locations within two of the deepest hadal sites on Earth, the Challenger Deep (10,918 meters below surface-mbs) and the Sirena Deep (10,667 mbs) within the Mariana Trench, as well as one control site (Ulithi Atoll, 761 mbs). Seawater samples were collected using an autonomous lander positioned ~1 m above the seafloor. The bacterial populations within the Mariana Trench bottom water samples were dissimilar to other deep-sea microbial communities, though with overlap with those of diffuse flow hydrothermal vents and deep-subsurface locations. Distinct particle-associated and free-living bacterial communities were found to exist. The hadal bacterial populations were also markedly different from one another, indicating the likelihood of different chemical conditions at the two sites. In contrast to the bacteria, the hadal archaeal communities were more similar to other less deep datasets and to each other due to an abundance of cosmopolitan deep-sea taxa. The hadal communities were enriched in 34 bacterial and 4 archaeal operational taxonomic units (OTUs) including members of the Gammaproteobacteria, Epsilonproteobacteria, Marinimicrobia, Cyanobacteria, Deltaproteobacteria, Gemmatimonadetes, Atribacteria, Spirochaetes, and Euryarchaeota. Sequences matching cultivated piezophiles were notably enriched in the Challenger Deep, especially within the particle-associated fraction, and were found in higher abundances than in other hadal studies, where they were either far less prevalent or missing. Our results indicate the importance of heterotrophy, sulfur-cycling, and methane and hydrogen utilization within the bottom waters of the deeper regions of the Mariana Trench, and highlight novel community features of these extreme habitats.

## Introduction

Hadal trenches are the deepest habitats on the surface of the Earth (Jamieson et al., 2010). Factors such as near-freezing temperatures, seafloor topography, subduction-linked physical and chemical features, and in particular, high pressures, are all likely to have contributed to the evolution and persistence of distinct microbial species (Simonato et al., 2006; Lauro and Bartlett, 2008), as has been more thoroughly documented for trench fauna (Blankenship and Levin, 2007; Jamieson et al., 2010, 2011). Despite the harsh conditions present in hadal trenches, microbial abundance and activity can excel there, at least within surficial sediments present along the trench axis where particulate organic carbon (POC) accumulates (Glud et al., 2013; Ichino et al., 2015).

At moderate depths in meso- and bathypelagic environments the vertical transport of particulate organic matter is a key driver of microbial activity, but estimates of this activity exceed the measured influxes of organic carbon, perhaps because of the importance of lateral advection and slowly sinking particles, as well as carbon fixation in these regions of the dark ocean (Burd et al., 2010). In most benthic settings the predominant factor influencing microbial abundance and activity is also the flux of POC (Moeseneder et al., 2012). These fluxes, together with reduced grazing pressure can produce prokaryotic cell abundances in the top regions of deep-sea sediments of more than 10^8^ cells cm^−3^, i.e., more than that which exists in productive surface waters (Jorgensen and Boetius, 2007; Boer et al., 2009; Schauer et al., 2010).

Detailed studies have been conducted of microbial assemblages present in bathypelagic and abyssopelagic zones and other specialized deep environments including hydrothermal systems, methane-dominated regions, and deep subsurface sediments (DeLong et al., 2006; Sogin et al., 2006; Martín-Cuadrado et al., 2007; Konstantinidis et al., 2009; Xie et al., 2011; Lauro and Williams, 2014). In contrast, most characterization of the microbes present in hadal environments has been pioneered using culturing or conventional 16S rRNA gene cloning and sequencing (Yayanos et al., 1979; Li et al., 1999; Yanagibayashi et al., 1999; Vezzi et al., 2005; Pathom-Aree et al., 2006; Kato, 2011; Cao et al., 2014). These studies, though valuable, have mostly focused on heterotrophic Gammaproteobacteria.

As sequencing techniques have improved, so have their applications in studying deep (Huber et al., 2007) and, more specifically, hadal microbiology. Eloe et al. (2011a,b) were the first to use next-generation metagenomics to better characterize deep-sea trench microbial communities, using samples collected from the top of the hadopelagic region of the Puerto Rico Trench (~6000 mbs). This study revealed the presence of large numbers of genes for porins, sulfatases, glyoxylate and dicarboxylate metabolism, and heavy metal resistance distributed among a community containing Acidobacteria, Actinobacteria, Bacteroidetes, Chloroflexi, Marinimicrobia, SAR11, and Planctomycetes as its major members, in addition to a very large proportion of Proteobacteria.

More recently, Nunoura et al. (2015) used tag sequencing and targeted gene PCR to vertically profile the microbial community of the Challenger Deep of the Mariana Trench, which at depths as great as 10,924 mbs represents the deepest region on Earth. With samples extending from surface waters down to 10,257 mbs, a clear shift in pelagic communities was discovered during the transition from abyssal to hadal zones, including an increasing number of heterotrophic taxa (primarily belonging to *Pseudomonas* and *Halomonas*) at the greatest depths. They hypothesized based on trench topography that the abundances of these heterotrophic microbes likely metabolized locally recycled organic carbon.

In this study we have extended the characterization of the microbiome of the Mariana Trench by sampling along the trench axis within the bottom waters of the Challenger and Sirena Deep, as well as at one relatively close comparison site located outside the trench. Illumina tag-based sequencing of the hypervariable V6 16S rRNA region was applied to 3.0, 0.22, and 0.1 μm filtered samples in order to discriminate among particle-associated, free-living and reduced cell size populations. The results provide new details of hadal bacteria and archaea and their relationships to prokaryotic assemblages occupying other deep-sea habitats.

## Materials and methods

### Sample collection

**Figure 1:**
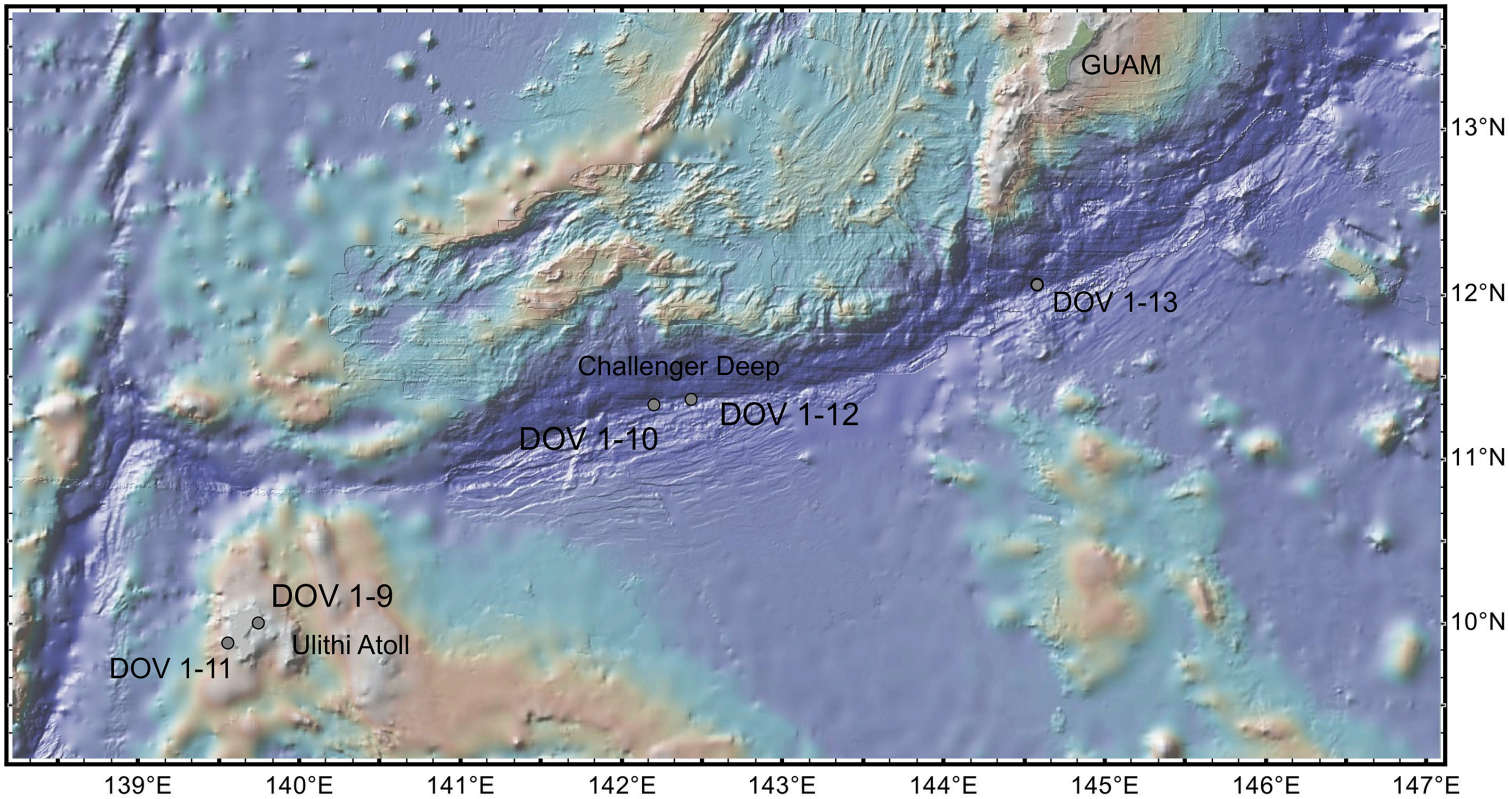
**M. T. sampling location**. The three sampling sites employed in this study are indicated by green circles overlaid onto a bathymetry map of the southwestern portion of the Mariana Trench. Points labeled DOV 1-9, 1-12, and 1-13 refer to Ulithi Atoll, Challenger Dee, and Sirena Deep sites, respectively.

Water samples and corresponding chemical data were collected from sites within the Challenger Deep (11.36902N 142.43294E, 10,918 mbs), the Sirena Deep (12.03924N 144.34868E, 10,667 mbs), and the Ulithi Atoll (10.00645N 139.74602E, 761 mbs; Figure 1). At each site, 60 L of seawater were collected using two 30 L lander-attached Niskin bottles resting approximately 1 m above the sediment surface (Hardy et al., 2014). Upon resurfacing, the bottles were emptied into chilled storage bins lined with autoclaved plastic Teflon storage bags for immediate dark filtration to prevent thermo- and photolysis. The seawater was then pumped using a peristaltic pump through a 3.0 (142 mm Supor), 0.22 (Sterivex cartridge), and 0.1 μm (142 mm Supor) filter series to collect large/particle-associated microbes, medium-sized free-living microbes, and putative nano-sized prokaryotes, respectively. Filters were stored in sucrose-Tris buffer (Fuhrman et al., 1988) at −20°C at sea and −80°C thereafter. The inorganic nutrient chemical analyses were performed at the Oceanographic Data Facility (ODF) at Scripps Institution of Oceanography. The procedures can be found at https://scripps.ucsd.edu/ships/shipboard-technical-support/odf/documentation/nutrient-analysis.

### DNA extraction/sequencing

Cell lysates were prepared from filters by manually crushing the frozen filters over dry ice, adding a 10% sodium dodecyl sulfate in NaCl-Tris-EDTA (SDS-STE) solution, and subsequent boiling as previously described (Fuhrman et al., 1988). DNA was then precipitated using separate sodium acetate and ethanol centrifugation steps followed by a phenol/SEVAG (CHCl_3_:Isoamyl alcohol 24:1 volume/volume) cleanup (1:1 mix, centrifugation, bottom layer removal). Samples were sent to the Marine Biological Laboratory (Woods Hole, MA) for sequencing. Bacterial 967F/1064R and archaeal 958F/1048R primers (Huber et al., 2007) were fused with Truseq adapter sequences for bridge amplification. After sequencing, reads were quality assessed using tag adapter and sequencing primer matches, adapting the pipeline suggested by Huse et al. (2007). These were then trimmed and quality filtered (Huse et al., 2014), and the datasets were uploaded to the Marine Biological Laboratory VAMPS (Visualization and Analysis of Microbial Population) database and website. Sequence data can be accessed at http://vamps.mbl.edu under the projects DCO_CMT_Av6 and DCO_CMT_Bv6.

### Sequence processing and classification

Bioinformatic analysis was performed using the MOTHUR v.1.33.3 program (Schloss et al., 2009). Sequences were downloaded from VAMPS, grouped uniquely, and aligned with the August 2013 version of the Greengenes reference alignment (DeSantis et al., 2006). Poorly aligned sequences (those sequences starting alignment after 99% of sequences) were removed and the remaining sequences were filtered for chimeric (Edgar et al., 2011) reads, then binned into operational taxonomic units (OTUs) with a similarity cutoff of 3%. OTUs were classified by assigning taxonomy at ≥80% similarity using the SILVA database v102 (Pruesse et al., 2007). Following sequence classification, those OTUs corresponding to common next-generation sequencing contaminants were removed (Laurence et al., 2014), except for *Pseudomonas* based on results from previous Mariana Trench studies (Kato et al., 1997; Nunoura et al., 2015). The general features of the Illumina-tag sequence datasets are listed in Table 1.

**Table 1:** **General features of the Illumina-tag sequence datasets**.

	**Sample**		**Number of sequences**	**Number of singleton reads**	**% Singleton reads**	**Number of OTUs**	**Number of un-classified OTUs**	**% Un-classified OTUs**	**% Un-classified**
Bacteria	Challenger	0.1 um	240,869	6132	2.55	9990	2496	24.98	8.13
		0.22 um	186,021	6137	3.3	10,351	3000	28.98	9.00
		3.0 um	333,553	5347	1.6	8585	2264	26.37	4.46
	Sirena	0.1 um	169,422	3303	1.95	5314	1552	29.21	3.54
		0.22 um	753,580	5783	0.77	6977	2773	39.74	2.2
		3.0 um	599,228	10,604	1.77	16,873	5526	32.75	7.77
	Ulithi Atoll	0.1 um	169,402	1751	1.03	2928	602	20.56	2.25
		0.22 um	171,044	4659	2.72	7502	2086	27.81	7.17
		3.0 um	237,207	5679	2.39	9036	2312	25.59	4.55
Archaea	Challenger	0.1 um	174,961	3838	2.19	5989	1621	27.07	4.17
		0.22 um	241,664	4085	1.69	6373	1979	31.05	5.03
		3.0 um	531,790	6183	1.16	10,357	3008	29.04	6.53
	Sirena	0.1 um	122,898	3039	2.47	4778	1270	26.58	7.83
		0.22 um	254,689	3402	1.34	5411	1542	28.5	3.48
		3.0 um	260,746	3803	1.46	5975	1924	32.2	4.82
	Ulithi Atoll	0.1 um	446,628	6371	1.43	10,151	2967	29.23	5.55
		0.22 um	1,092,348	6871	0.63	11,579	3146	27.17	3.91
		3.0 um	292,921	4480	1.53	6972	2120	30.41	4.07

### Alpha and beta diversity

To compare our samples and other relevant marine communities, we analyzed our dataset with other V6 tag-sequenced marine datasets downloaded from the VAMPS database. Site-specific evenness, diversity, and richness measurements were determined by the summary.single command in MOTHUR. Evenness was calculated using the Simpson evenness index. Diversity was calculated using Inverse Simpson and Shannon indices, and richness measurements were determined using the Chao richness calculator. All comparative diversity measurements were normalized to 90% of the smallest sized dataset by 1000 iterations of subsampling. Similarity trees were calculated using the MOTHUR tree.shared command, specifying a Yue-Clayton similar distance matrix (Yue and Clayton, 2005). Finally, comparative heatmaps were made using the multiple group heatmap comparison function of the STAMP bioinformatics package (Parks et al., 2014).

### Curation of deep-enriched taxa

The datasets were manually curated for taxa enriched in hadal samples by dividing relative abundances of OTUs at depth with their corresponding shallow reference samples from the Ulithi Atoll abundances in comparable filter sizes, and removing sequences with a ratio <1. Only those OTUs showing a relative abundance of 0.01% or greater were used for this analysis, similar to cutoffs used by other V6 16S rRNA gene sequence analyses using the same sequencing platform (Galand et al., 2009a).

## Results

Nepheloid layer water samples were obtained from the Challenger Deep and Sirena Deep within the Mariana Trench and from a shallower deep-sea control site at the Ulithi Atoll. General nutrient and sampling location data is described in Table 2. The temperature and silicate concentrations suggest that the source of the seawater in all three locations is Lower Circumpolar Deep Water, consistent with information indicating that Southern Ocean-sourced Lower Circumpolar Deep Water enters into the Mariana Trench from the north and exits into the West Mariana Basin and the Yap Trench at the Mariana Trench-North Yap Escarpment-Parece Vela Rift apparent triple junction (Fujiwara et al., 2000; Ohara et al., 2002). Particle-attached microbes, free-living microbes and reduced-size free-living microbes were collected onto 3, 0.22, and 0.1 μm in-line filters, respectively. This material provided the source of DNA for taxonomic study based on tag-sequencing and subsequent bioinformatics analyses. A total of 2,860,326 bacterial and 3,418,645 archaeal sequences were obtained. The composition of the prokaryotic communities was assessed and compared with other VAMPS marine datasets compiled using the same sequencing platform. Approximately 5% of both the bacterial and archaeal sequences were unclassifiable when compared with the reference databases employed.

**Table 2:** **Sampling site chemical and location data**.

**Location name**	**Lat/Long**	**Depth (m)**	**Temp (°C)**	NO_3_ + NO_2_μM^*^	**PO_4_μM**	**Silicate μM**	**NH_4_μM**
Ulithi Atoll region	10.00645N 139.74602E	761	6	32.45	2.29	92.3	0.20
Challenger Deep	11.36902N 142.43294E	10,918	2.5	29.255	2.42	106.9	0.47
Sirena Deep	12.03924 N 144.34868E	10,677	2.5	29.14	2.295	106	0.15

* *NO_2_ values below 0.01 μM*.

### Diversity measurements

**Figure 2:**
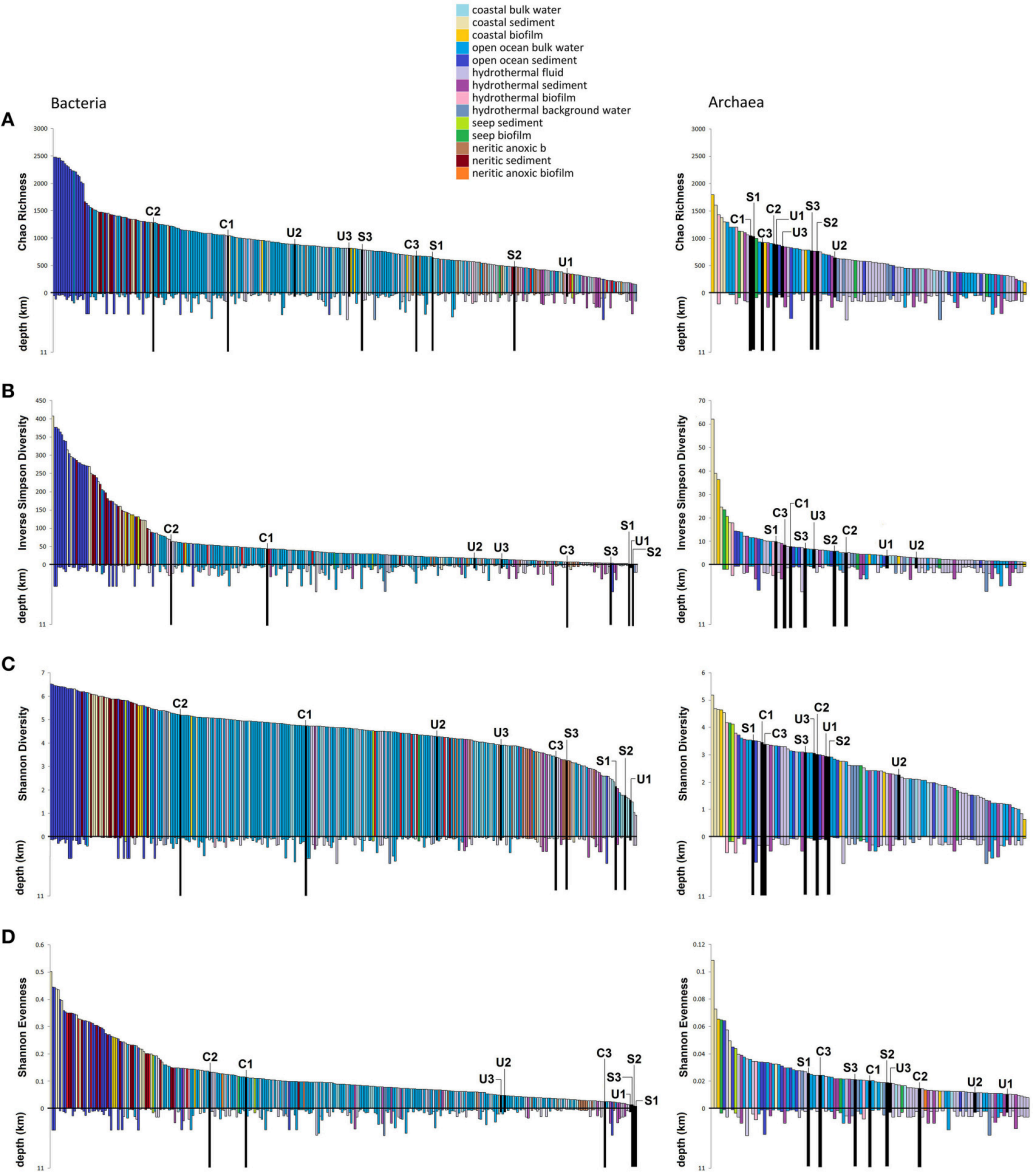
**Comparative richness, diversity, and evenness of different V6-sequenced marine environments**. Chao richness **(A)**, Simpson evenness **(B)**, Inverse Simpson, and Shannon diversity/ evenness **(C,D)** of each size-fractionated Mariana Trench/Ulithi Atoll sample as well as different VAMPS datasets are shown. Values were calculated with the summary.single MOTHUR command using 1000 iterations of bootstrapped subsampling (at 90% of the smallest dataset in the analysis). Samples are color coded based on habitat, with bars below the X axis representing sample depth. Samples from our study are colored in black. Symbols C, S,U and 1, 2, 3 represent Challenger Deep, Sirena Deep, Ulithi Atoll and 0.1, 0.22, and 3.0 μm, respectively.

We used Chao richness, inverse Simpson/Shannon diversity, and Simpson evenness (Shannon, 1948; Simpson, 1949; Chao, 1984) indices to compare different aspects of alpha diversity of the Mariana Trench and Ulithi Atoll microbes with those of other marine datasets. Bacterial communities from our samples were mostly low in relative diversity, except for the Challenger Deep free-living fractions, which showed moderate to high richness/diversity, comparable with other open-ocean samples (Figure 2). Sirena Deep communities were particularly lacking in diversity, but possessed higher relative richness. Overall, archaea within all samples were much less diverse than bacteria (though relatively diverse compared with other archaeal datasets) and showed less variation based on sampling site and more variation based on filter pore size. The Ulithi Atoll archaea were among the least diverse (Figure 2).

### Community comparisons

**Figure 3:**
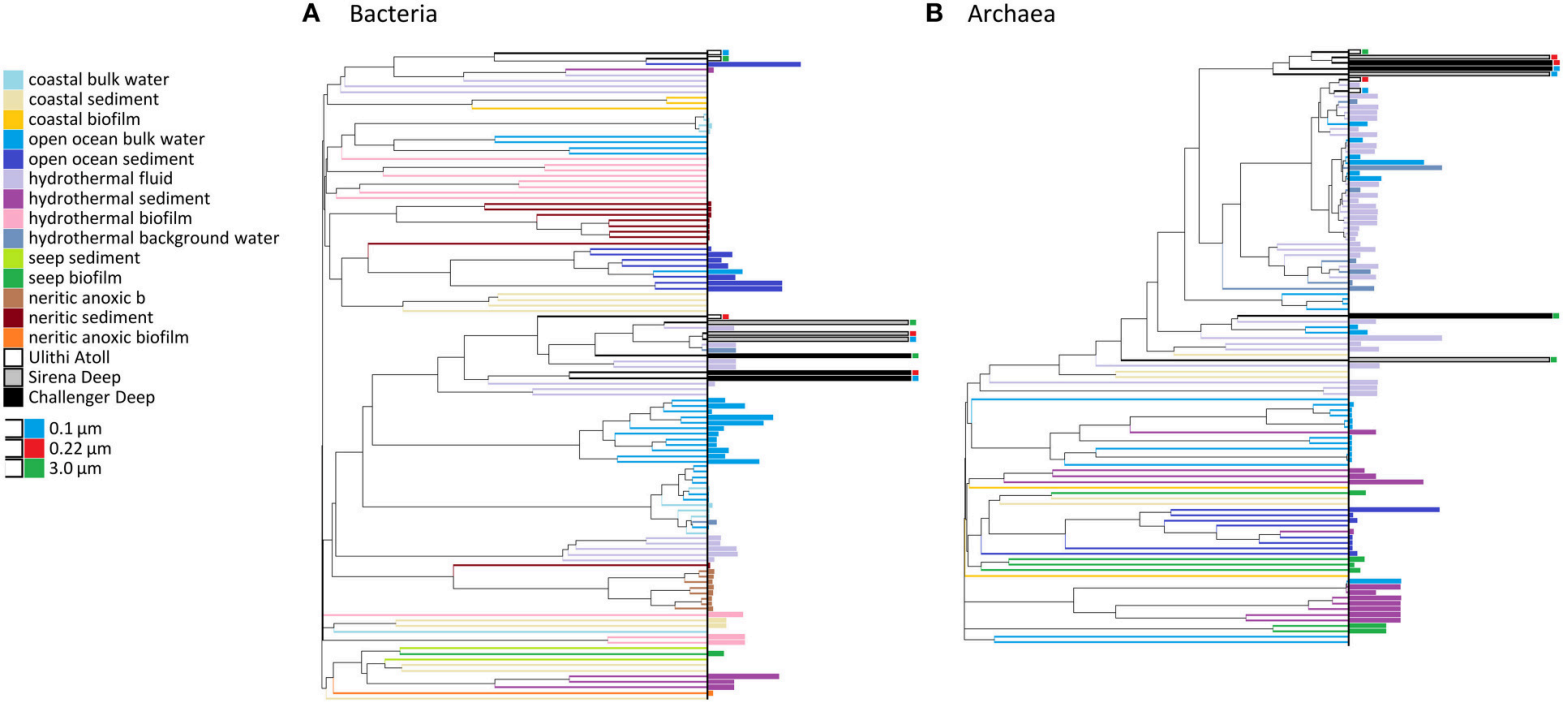
**Similarity trees of different marine datasets of bacteria (A) and archaea (B)**. VAMPS V6 datasets were compared with Mariana Trench communities using a Yue-Clayton calculated similarity matrix made by the tree.shared command in MOTHUR. Sampling site habitat is represented based on color and vertical distance from the end of the branch represents depth of sample. Samples from our study are labeled with colored rectangles reflective of the pore size of the filter.

The microbial communities in all three of our sampled sites were compared with other marine communities using Yue-Clayton derived similarity trees, which takes into account the proportions of the taxonomic groups present (Kato et al., 1997). At the OTU level, the bacterial assemblages within the Mariana Trench samples predominately clustered by collection site and by filter size, with free-living fractions grouped separately from particle-associated communities. They were also notably placed within a clade that also included low-temperature diffuse flow hydrothermal communities from the Axial seamount (from 2 to 49°C; Figure 3). Both the 3.0 and 0.1 μm Ulithi Atoll filter fractions grouped with deep oceanic sediment and shallow vent sites while the 0.22 μm filter clustered alongside the Mariana Trench samples. The separate grouping of the 3.0 and 0.1 μm Ulithi Atoll fractions was the result of a relatively low number of Gammaproteobacteria and an increased abundance of Alphaproteobacteria.

**Figure 4:**
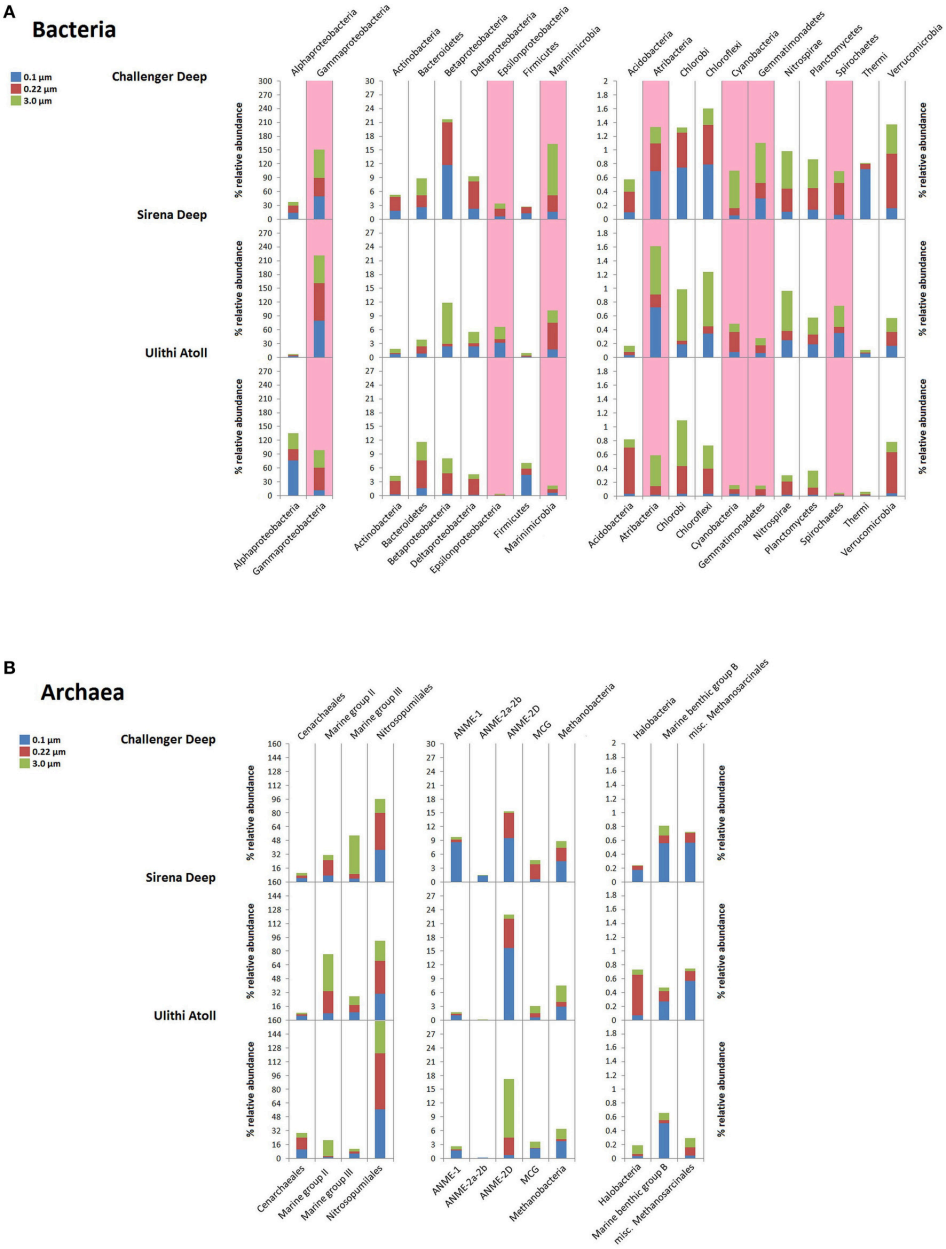
**Major microbial taxa represented in Mariana Trench bacterial (A) and archaeal (B) samples**. Groups bearing a relative abundance of 0.1% or greater (within at least one sample) are shown. Filter sizes corresponding to the same sample site are represented as vertically adjacent. Bars are laid out to a possible maximum of 300% relative abundance–100 from each filter, stacked vertically. Graphs are arranged from left to right based on different scales of abundance from high to low. Taxa highlighted in pink show depth-enrichment across all hadal sites.

Similar to the Mariana Trench bacterial samples, archaeal samples clustered alongside hydrothermal-associated datasets. There was a clear separation between particle-associated and free-living archaeal communities resulting from the enrichment of groups of Euryarchaeota on particles (Figure 4).

### Site-specific communities

#### Challenger deep

The Challenger Deep bacterial community was highlighted by a diversity of abundant groups, most notably Gammaproteobacteria (Figure 4). Differences between particle-associated and free-living fractions as seen in diversity measurements and similarity trees were reflected in taxa abundance. Betaproteobacteria, Deltaproteobacteria, Chlorobi, Atribacteria, and Chloroflexi were prevalent among free-living bacteria, whereas Marinimicrobia, Cyanobacteria, Gemmatimonadetes, and Nitrospirae (largely Thermodesulfovibrionaceae) were more abundant in the particle-associated filter. Alphaproteobacteria were also abundant across all Challenger Deep filter samples, with OTUs from Rhizobiales, Rhodobacteriaceae, and Pelagibacteriaceae enriched relative to other sampling sites. Notable Challenger Deep enriched OTUs included those of Marinimicrobia and *Oleibacter*. The free-living bacterial fraction also contained previously cultured piezophiles (high pressure-adapted microbes), including V6 sequences identical to the isolates *Colwellia* strain KT27, *Colwellia piezophila* and *Moritella abyssi* (accounting for 0.23, 0.1, and 0.31%, of the bacterial population, respectively). These relative abundances of classifiable piezophiles are higher than those in the Puerto Rico Trench, in which unclassified Psychromonadaceae and Shewanellaceae sequences were found but in low numbers (Eloe et al., 2011b). In other hadal datasets, putatively piezophiles were altogether absent.

Among archaea, the free-living fraction was dominated by *Nitrosopumilus* and anaerobic methane-oxidizing archaea (ANME-1 and 2D), with *Nitrosopumilus* OTUs representing over 45% of both the 0.1 and 0.22 μm filters (Figure 4). The Challenger Deep particle-associated fraction was enriched in Marine Group II and Marine Group III (Euryarchaeota) and Marine Benthic Group A archaea.

#### Sirena deep

The Sirena Deep bacterial profile possessed low diversity resulting from the dominance of a limited number of OTUs belonging to the Gammaproteobacteria (Figure 4). This group was particularly enriched in the free-living 0.22 μm filter fraction, where Gammaproteobacteria together with Marinimicrobia accounted for over 80% of the total community (Figure 4). The Sirena Deep bacterial community also had sizeable fraction of deep-subsurface associated OTUs based on BLAST alignments. These included *Desulforudis*, Atribacteria, Epsilonproteobacteria, Deltaproteobacteria, Betaproteobacteria, and numerous unclassified OTUs (which together combined for up to ~19% of the population).

The archaeal assemblages in the Sirena Deep were similar to that of the Challenger Deep samples. *Nitrosopumilus* and Euryarcheaota were highly abundant in free-living and particle-associated fractions, respectively, though the dominant Euryarchaeota sequences between sites differed: Marine Group II Euryarchaeota were dominant in the Sirena Deep and Marine Group III Euryarchaetota were more numerous in the Challenger Deep. Anaerobic methane-oxidizing taxa were not as prevalent in Sirena Deep communities, though ANME 2-D sequences were numerous.

#### Ulithi atoll

The Ulithi Atoll community was dominated by Alphaproteobacteria and *Nitrosopumilus* (Figure 4). Among the Alphaproteobacteria, by far the most abundant OTU within the sample belonged to the *Erythromicrobium-Porphyrobacter-Erythrobacter* group, totaling up to 69.5% of the 0.1 μm bacterial community and on average being the most abundant taxon across all three filter sizes.

### Hadal-enriched taxa

**Figure 5:**
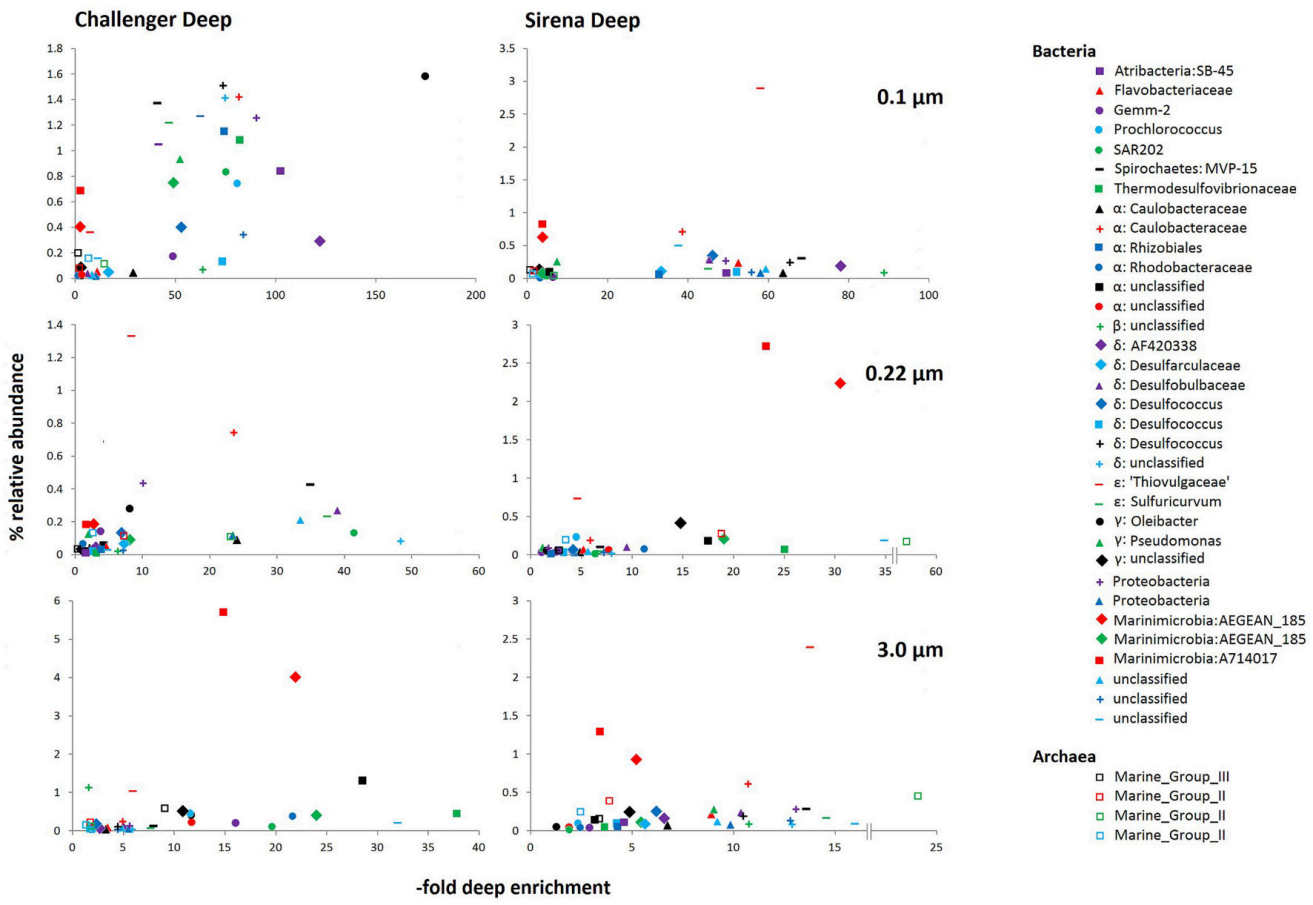
**Hadal-enriched OTUs**. Only OTUs with a relative abundance of 0.01% across all hadal sites were plotted. Within bacteria, singular taxa were listed first. Archaea are shown as blank squares.

Hadal-enriched taxa were determined by selecting those OTUs that were enriched across the Challenger Deep and Sirena Deep trench filters relative to the Ulithi Atoll abundances. Thirty four bacterial and four archaeal OTUs were preferentially found in the hadal sampling sites (Figure 5). The most abundant of these OTUs belonged to Marinimicrobia and Epsilonproteobacteria, while less abundant taxa included Atribacteria, Caulobacteraceae, Cyanobacteria, Deltaproteobacteria, Gemmatimonadetes, *Oleibacter*, and Spirochaetes. All of the four hadal-enriched archaea OTUs were Euryarchaeota (three Marine Group II and one Marine Group III, Figure 5).

## Discussion

### Challenger deep community

Bacteria in the Challenger Deep consisted largely of general seawater microbes including Gammaproteobacteria, Marinimicrobia, and *Pelagibacter*, as well as the cyanobacterium *Prochlorococcus*, and was far more diverse and rich than the Sirena Deep. It therefore seems that Mariana Trench bacterial communities are relatively heterogenous between different sample sites. As with the Sirena Deep, there were no major differences in dominant OTUs between the free-living Challenger Deep bacterial filter fractions. This was reflected in the high Yue-Clayton similarity between the 0.1 and 0.2 μm groups compared with other surveyed datasets (Figure 3). Thus, it appears that many bacteria within the sites examined exhibit a range of sizes that extend down to dimensions smaller than those generally considered in microbial oceanography. The free-living fraction also contained high counts of Betaproteobacteria. By far the three most abundant betaproteobacterial OTUs across our datasets were assigned taxonomies *Alcanivorax*, Thiobacteraceae, and an unclassified Betaproteobacteria sequence that was found in a multitude of deep subsurface samples. Combined, these groups accounted for more than a third of the local Betaproteobacteria abundance in both trench samples.

In contrast, archaea were more similar across different hadal sites, consistent with other studies of pelagic archaea showing that a few cosmopolitan marine taxa make up the majority of open ocean archaea (Massana et al., 2000; Anderson et al., 2015). In our samples, *Nitrosopumilus* was particularly dominant, as seen in other deep studies (Vetriani et al., 1999; Massana et al., 2000; Hu et al., 2011; Park et al., 2014). Furthermore, our data showed possible niche partitioning of major archaeal groups, with *Nitrosopumilus* being the prevalent taxa in free-living samples and Euryarchaeotal sequences dominating particle-associated fractions. The observed separation between particle-associated and free-living archaeal communities contrasted with other deep-sea size-fractionated archaeal community studies that showed a more homogenous distribution of taxa between particle-attached and free-living populations (Galand et al., 2009b; Eloe et al., 2011b; Smith et al., 2013), though other examples of distinct attached and free-living archaeal communities do exist (Wells and Deming, 2003; Orsi et al., 2015).

Curiously, many of the minor groups of Archaea appear to be enriched in the 0.1 μm fraction (Figure 3). However, the patterns of abundance for these Archaea generally mirror those of the 0.2 filters down to the OTU level. This apparent “enrichment” may be explained by the dramatically decreased dominance of MG-II and MG-III sequences in the 0.1 μm sample, resulting in an overall increase in abundances of other OTUs. However, it is worth noting that at least one of these taxa, the Marine Benthic Group B Archaea, is known to contain members with reduced cell size (Knittel et al., 2005). As very little is known about the ecology of these uncultivated archaeal groups, more research on this topic is needed.

The discovery of abundant OTUs in the Challenger Deep matching to known piezophiles from the genera *Colwellia* and *Moritella* contrasts with other culture-independent hadal studies (Eloe et al., 2011a,b; Nunoura et al., 2015), where comparable sequences were less than one third of those found in our samples. As expected, most of these sequences showed enrichment in hadal samples relative to their abundances in the Ulithi Atoll. These results provide the first information on a site where culturable piezophiles identified at the genus level are a notable portion of a hadal community.

### Sirena deep community

The close relatedness of Mariana Trench communities to hydrothermal vent samples including background fluids from several Mariana back-arc magma volcanoes (Figure 3) was most apparent in the Sirena Deep bacterial samples. Taxa shared between our samples and vent datasets include Gammaproteobacteria, SAR324, Marinimicrobia, Thiovulgaceae, and SUP05, all of which are frequently encountered in vent datasets. Such relationships might be explained by the similarities between microbes associated with marine organic particles and entrained vent plumes (Dick et al., 2013). Anoxic particle surfaces can give rise to these vent-related microbial taxa, as previously seen in the Japan Trench following the Tohoku earthquake (Kawagucci et al., 2012). However, the high abundance of deep subsurface-associated OTUs within these taxa may also reflect connectivity among Mariana Trench and surrounding regions containing seamounts, serpentine seeps, and mud volcanoes (Fryer et al., 1985; Roth and Dymond, 1989; Gamo et al., 2004; Mottl et al., 2004; Ohara et al., 2012; Feseker et al., 2014).

Using BLAST-directed search of our sequences, we found that OTUs in the Sirena Deep showed a high abundance of deep-subsurface associated taxa. Among these were OTUs of the genus *Desulforudis*, which were 1.9% of the particle-associated fraction and were absent from 322 other VAMPS datasets outside our study. This genus has previously been found deep underground in depths as great as 3 km below the surface as well as subsurface basement ridge fluids, where it is believed to exist through hydrogen oxidation couple with sulfate reduction (Chivian et al., 2008; Jungbluth et al., 2013). The taxonomic features of the microbes present in the bottom waters of the Sirena Deep suggest that it could be a reducing environment, consistent with other studies (Hand et al., in preparation).

### Ulithi atoll community

Dominant taxa in the Ulithi Atoll community appeared to be consistent with its comparatively shallow profile. Of these taxa, facultative anoxygenic phototrophs of the *Erythromicrobium-Porphyrobacter-Erythrobacter* group were especially numerous. These bacteria are common fixtures of neritic, organic-rich marine sediments, with many possessing considerable hydrocarbon-degrading abilities (Roling et al., 2002; Goodwin et al., 2005; Alonso-Gutiérrez et al., 2009). Related genera have been also observed in association with deep-sea polymetallic nodules and hydrothermal vent plumes (Yurkov et al., 1999; Xu et al., 2009). Flavobacteria and Kiloniellaceae were also abundant in the Ulithi Atoll samples. The former taxa is widespread in coastal marine environments (Buchan et al., 2014), while the latter is known primarily from a marcoalgal isolate (Wiese et al., 2009).

### Major hadal microbial taxa

Among the hadal-enriched taxa present in our samples, several are also abundant in other hadal datasets, particularly Marinimicrobia and Gemmatimonadetes (Eloe et al., 2011b; Nunoura et al., 2015). Additionally, when examining the abundance of hadal-enriched OTUs across the VAMPS V6 datasets, Marinimicrobia, and Gemmatimonadetes sequences showed consistent deep enrichment. Both maintain high overall abundances in communities below 4000 mbs (projects KCK_NADW, KCK_SMT). This was also seen in our hadal-enriched archaeal OTUs, with the most abundant OTU being almost 10% of the community in a 4988 mbs microbial mat adjacent water sample from the Loihi Seamount. The strong showings of these groups in hadal and deep abyssal datasets support their importance in extremely deep environments and suggest that they are likely part of a conserved abysso/hado-pelagic microbial community.

#### Gammaproteobacteria

**Figure 6:**
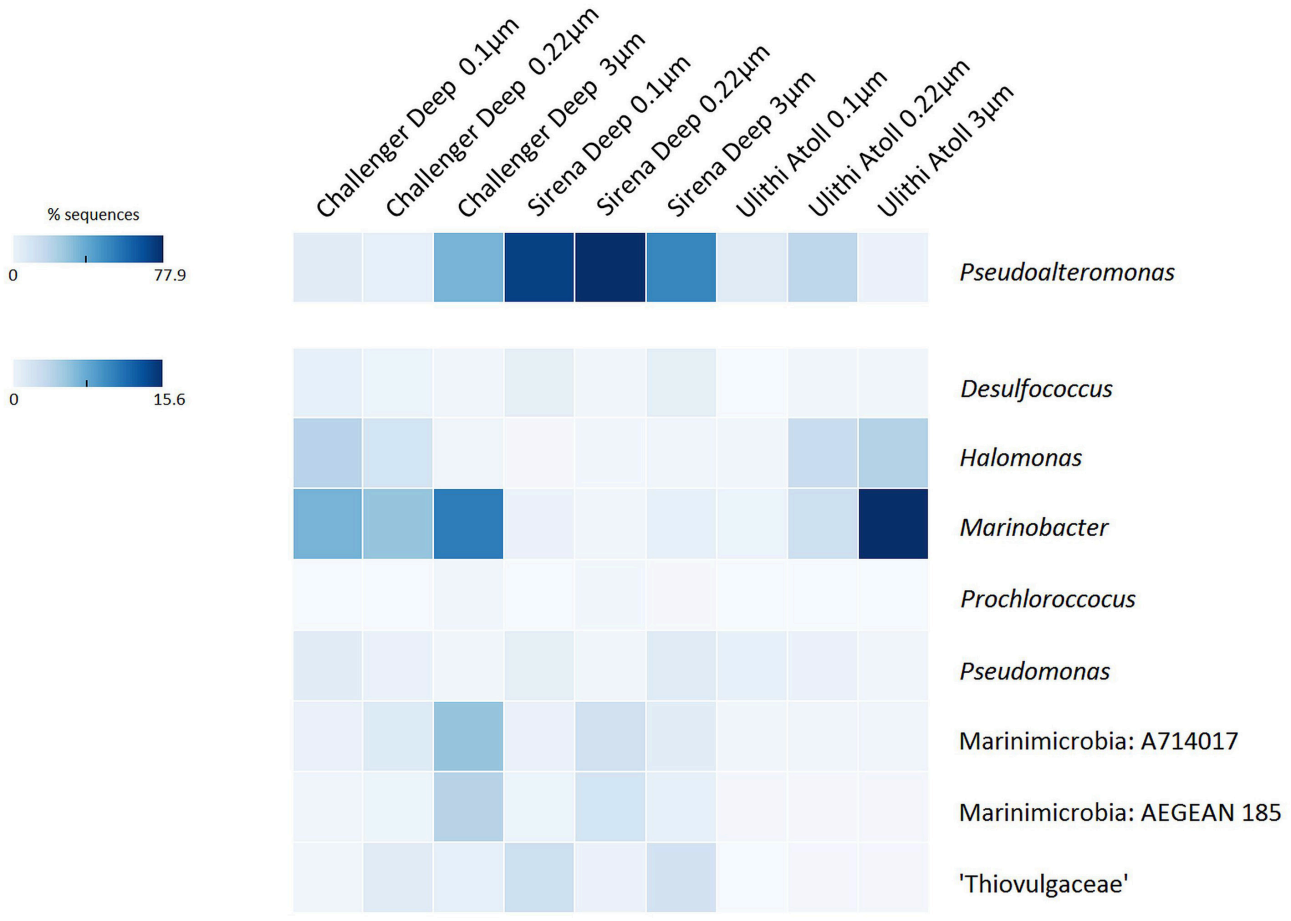
**Heatmap of Mariana Trench Bacterial Taxa**. A heatmap of taxa of interest (highly abundant and major hadal-associated taxa) was created using the multiple group heatmap comparison function of STAMP bioinformatics package (Parks et al., 2014). Depth of color indicates percent abundance based on keys to the left. *Pseudoalteromonas* was separated from other taxa due to higher scale of abundance.

Gammaproteobacteria primarily of the cosmopolitan marine genera *Pseudoalteromonas, Alteromonas*, and *Marinobacter* were clearly the most dominant taxa in Mariana Trench microbial communities (Figure 6). These genera have been identified in deep-sea vent sequences and isolates (Ivars-Martinez et al., 2008; Baker et al., 2013; Campbell et al., 2013; Kato et al., 2013; Lekunberri et al., 2013), as well as hydrothermal background waters and plumes (Sylvan et al., 2012; Bennett et al., 2013; Dick et al., 2013; Sheik et al., 2015). *Pseudoalteromonas* were especially abundant in the microbial communities of the Sirena Deep, and are commonly represented in deep-sea datasets (Radjasa et al., 2001; Cui et al., 2008; Dong et al., 2015). They have also been cultured in high numbers from Sirena Deep sediments (Logan et al., unpublished data) and other deep-ocean sites. High abundances of *Pseudoalteromonas* have previously been observed in seamount background water, hydrothermal fluids, and vent flocculent material (Meyer et al., 2013; Figure 2), and were largely responsible for the co-clustering of our hadal samples with diffuse flow hydrothermal datasets. In deep-sea pelagic datasets, they are far less numerous. Members of this genus recovered from hydrothermal vent environments are metal-resistant and are capable of sulfur oxidation (Teske et al., 2000; Holden and Adams, 2003; Nichols et al., 2005; Carvalho, 2013; Li et al., 2014).

Gammaproteobacteria enrichment in the deeper waters of the Mariana Trench was also noted by Nunoura et al. (2015) in vertical profiles of the Challenger Deep. From their abundance data, they suggested that heterotrophy is enriched at the greater depths of the Challenger Deep due to the retention of organic matter within the slowly ventilating steep-walled enclosure provided by the trench, coupled with its resuspension into the overlying waters as a result of earthquake activity. We also inferred high levels of heterotrophy in both the Challenger Deep and the Sirena Deep based on the abundances of Gammaproteobacterial sequences, though we found a more varied and diverse group of these microbes in the Challenger Deep than that reported by Nunoura et al. (2015). These differences could stem from the greater influence of surficial sediments on our samples (Turley, 2000).

#### Sulfur-cycling taxa

Sequences of both sulfur-oxidizing and sulfur-reducing microbes were recovered in high numbers from the Mariana Trench (Figures 5, 6). Sulfur cycling is known to be an important process in the deep-sea, particularly in deep sediment horizons, seeps, and hydrothermal environments, and is thought to contribute significantly to heterotrophy (sulfur reduction) and chemolithoautotrophy (sulfur oxidation) in these environments.

By far the most abundant sulfur-cycling OTU in our samples belonged to metabolically plastic sulfur-oxidizing Epsilonproteobacteria of the *Sulfurovum-Sulfurimonas* group (family nov. “Thiovulgaceae”), most prevalently in the Sirena Deep. These organisms are widespread in vent chimney biofilms, seep sediments, and invertebrate microflora (Inagaki et al., 2003; Campbell et al., 2006; Mino et al., 2014). Within deep-sea sediments, particularly at the oxic-anoxic boundary and the sulfidic chemocline, these bacteria may provide a major source of localized carbon fixation. Surprisingly, our particular sequences matched to microbial mat streamers found in sulfidic, low-oxygen cave waters, where these taxa also persist (Macalady et al., 2008; Jones et al., 2010). Another prominent group of hadal sulfur oxidizers present in both of our deep samples sets belonged to SUP05 (Thiobacteriaceae), which are extremely common in hydrothermal-influenced seawater (Dissanayake et al., 2014). Energy sources such as reduced sulfur compounds, hydrogen and methane are primary determinants of microbial community composition in many deep-sea environments (Inagaki et al., 2003; Mino et al., 2014), as opposed to temperature, which exerts a greater influence on population structures in shallower epipelagic waters (Jones et al., 2010).

Among the sulfate reducers, *Desulfococcus* was the most common taxon (Figure 5). This genus is known for mediating the oxidation of a variety of hydrocarbons, including alkanes, alkenes, and aromatics (Kleindienst et al., 2014). Within the deep sea, *Desulfococcus* species associate with specialized hydrocarbon-rich environments including seeps, hydrate beds, and vents. As previously noted, the subsurface-associated sulfate reducing taxa *Desulforudis* (Thermodesulfovibrionaceae) was also abundant.

#### Cyanobacteria

Cyanobacteria, in particular, *Prochlorococcus*, were well represented in the hadal sites relative to the Ulithi Atoll. They were also common in several other deep-sea VAMPS datasets from the deep North Atlantic and Loihi and Forecast vent bottom water. Relatively high abundances of Cyanobacteria at depth have been previously observed, and are believed to play an important ecological role by delivering reduced carbon, often rapidly, to the dark ocean, thereby stimulating deep-sea heterotrophic activity (Thiel et al., 1989; Agusti et al., 2015). Cyanobacteria in the Mariana Trench are presumably also associated with sinking particles derived from upper water strata (Lochte and Turley, 1988; Glud et al., 2013). Evidence of phytodetritus was previously observed in the Mariana Trench, both in seawater (Nunoura et al., 2015) and sediment (Gooday et al., 2010). Additionally, other hadal datasets also show similar abundances of Cyanobacteria (Eloe et al., 2011b). Hadal enrichment of this taxon is believed to come from particulate organic matter focusing along the axis of the trench (Ichino et al., 2015; Nunoura et al., 2015), particularly beneath oligotrophic waters where they make up a greater portion of sinking phytodetritus (Agusti et al., 2015).

#### Marinimicrobia

OTUs from the Marinimicrobia, including three among the hadal-enriched members, were very abundant in Challenger Deep and Sirena Deep communities (Figures 4–6). This candidate phylum has low representation in non-deep VAMPS samples but high abundance in many deep pelagic datasets. Although it is often positively correlated with low dissolved oxygen (Pham et al., 2008; Ghiglione et al., 2012; Allers et al., 2013; Parsons et al., 2014), little is known about its ecology or metabolic function. Representatives from both Marinimicrobia families were among the more abundant sequences recovered by multiple groups in the Mariana Trench (León-Zayas et al., 2015; Nunoura et al., 2015) and were also common in the Puerto Rico Trench (Eloe et al., 2011b).

#### Marine group II/III euryarchaeota

Members of the widespread pelagic Marine Group II and Marine Group III Euryarchaeota were enriched in the hadal particle-associated samples (Figures 4, 5). Surveys of these archaea from other eutrophic environments have also suggested important heterotrophic roles for these microbes on surfaces at depth (Orsi et al., 2015).

Marine Group III OTUs dominated the particle-associated sequences in the Challenger Deep (Figure 4). While Marine Group III Euryarchaeota are common members of the deep bulk water microbial community (Martin-Cuadrado et al., 2008; Galand et al., 2009b), functional and ecological information on this taxon is sparse. The most abundant Marine Group III OTUs from the Challenger Deep shared greatest similarity to NCBI sequences recovered from a wide range of deep sampling sites including gas hydrates, marine snow, crustal fluids, and abyssal seawater.

Particle-associated archaea populations in the Sirena Deep were dominated by Marine Group II Euryarchaeota. Water column vertical profiles often indicate highest numbers of Marine Group II reads at the sea surface (Massana et al., 1997, 2000; DeLong et al., 1999; Pernthaler et al., 2002), and this trend was also seen in other VAMPS communities. They are, however, also abundant in many deep datasets (López-García et al., 2001; Bano et al., 2004; Baker et al., 2013), even showing depth-dependent ecotype separation, with proteorhodopsin genes only being present in shallow-water members (Frigaard et al., 2006; Alonso-Sáez et al., 2011; Martin-Cuadrado et al., 2014). The top three most abundant Marine Group II sequences in the Sirena Deep were in lower abundance in the Challenger Deep (Figure 4) and not encountered in other VAMPS communities. They also showed relatively poor alignment and coverage with most group II sequences in the NCBI database save for one sequence from deep sediment basement fluids (Jungbluth et al., 2013). Thus, it appears that the particle-associated Sirena Deep microbial communities are dominated by novel groups of Euryarchaeota.

## Summary

The benthic boundary layer bottom water microbial communities of two hadal regions of the Mariana Trench have been shown to be surprisingly distinct, particularly among the bacteria. However, both deep trench sites were dominated by heterotrophic Gammaproteobacteria, as well as *Nitrosopumilus* and Euryarchaetoa. Other abundant taxa included Alpha- Beta- Delta- and Epsilonproteobacteria and anaerobic methane oxidizing archaea. Bacterial and archaeal communities in our samples showed different patterns of enrichment, with bacteria typically clustering based on sampling site and archaea according to the filter pore size fraction. Many of the analyses indicate connections between the Mariana Trench hadal samples and subsurficial and vent-derived communities and taxa. This was most evident in the case of the Sirena Deep, which harbored a distinct *Pseudoalteromonas*-dominated microbial community. Clear patterns of hadal enrichment were identified in specific OTUs belonging to a number of phyla and subphyla, including Gammaproteobacteria, Epsilonproteobacteria, Marinimicrobia, Cyanobacteria, Deltaproteobacteria, Gemmatimonadetes, Atribacteria, Spirochaetes, and Euryarchaeota. Additional studies will be needed to assess the significance of the microbes identified to biogeochemical processes occurring at great depth as well as the generality of our results over time and in additional hadal locations.

## Author contributions

JT analyzed the primary datasets and was the main contributor to scientific synthesis. LP helped with data analysis and interpretation. KH developed sampling instrumentation and directed sample recovery and participated in data analysis discussions. JC provided expedition oversight and participated in data analysis discussions. DB provided science direction, contributed to data interpretation, and provided manuscript editing.

### Conflict of interest statement

The authors declare that the research was conducted in the absence of any commercial or financial relationships that could be construed as a potential conflict of interest.
